# Early Impact of the 2014 World Health Assembly Resolution on Palliative Care: A Qualitative Study Using Semistructured Interviews with Key Experts

**DOI:** 10.1089/jpm.2019.0384

**Published:** 2020-12-18

**Authors:** José Miguel Carrasco, Hamilton Inbadas, Alexander Whitelaw, David Clark

**Affiliations:** ^1^ APLICA Investigación y Traslación, Soc. Coop. Mad, Madrid, Spain.; ^2^ School of Interdisciplinary Studies, University of Glasgow, Scotland, United Kingdom.; ^3^ ATLANTES Research Programme, Instituto Cultura y Sociedad, University of Navarra, Navarra, Spain.

**Keywords:** advocacy, implementation, palliative care, public health, resolution, World Health Assembly, World Health Organization

## Abstract

***Background:*** In 2014, the World Health Assembly (WHA) approved the Resolution “*Strengthening of palliative care as a component of comprehensive care throughout the life course”* (WHA67.19), urging national governments to carry out actions to develop palliative care.

***Objective:*** To establish the origins and assess the influence and early impact of this Resolution.

***Methods:*** Semistructured interviews conducted with key informants (*n* = 20). A thematic content analysis was conducted and triangulated on the transcriptions.

***Results:*** The collaborative work done by Non-State Actors, palliative care associations, individuals, Member States, and the World Health Organization (WHO) itself was crucial to the drafting and the approval of WHA67.19. Several post-Resolution actions undertaken by the WHO were highlighted (e.g., appointment of a dedicated officer and the creation of advisory working groups) and its role was identified as a key element in the implementation. Inadequate funding, lack of resources, and cultural factors were the most relevant barriers to implementation. The wide network of NGOs and palliative care associations was identified as the main facilitator. The key identified impact of the Resolution was its value as an advocacy tool and its contribution to raising awareness about palliative care around the world.

***Conclusions:*** Despite the lack of indicators to monitor the implementation of Resolution WHA67.19, key experts evaluate its effects in the short term as positive. Policy potential and its use in championing palliative care are its main early successes. The role of Non-State Actors, the WHO, and Member States working together is crucial to achieving its goals.

## Background

Numerous sources highlight the importance of developing and integrating palliative care within health care systems, yet the evidence indicates that the supply of palliative care services is still hugely insufficient to meet the needs of the global population.^1–6^

In January 2014, the World Health Organization (WHO) Executive Board prepared a Resolution calling for action on the limited availability of palliative care in most of the world, the avoidable suffering of millions of people and their families, and the need to create or strengthen health systems that include palliative care as an integral component of treatment.^7^ The Resolution WHA67.19-*Strengthening of palliative care as a component of comprehensive care throughout the life course*—was approved in the 67th World Health Assembly (WHA), which took place in Geneva (Switzerland, May 19–24, 2014), urging Member States and requesting the WHO to carry out key actions to develop palliative care globally.^7^

Our objective was to establish the origins and to learn about the influence and early impact of Resolution WHA67.19 on the global development of palliative care.

## Study Design and Methods

We conducted semistructured interviews with key informants in “elite” positions among senior management or at board level within relevant stakeholder organizations involved in global palliative care development.^8^ Twenty people speaking in their own name and not on behalf of their institutions were interviewed (Appendix Table A1), from April to July 2017, following an interview guide based on a previous scoping review carried out on the topic. A thematic content analysis triangulated by two experienced researchers in public health (J.M.C.) and palliative care (H.I.) was carried out on the transcriptions.

## Results

### Origin, preparation, and adoption

When participants were asked how and why the Resolution came about, most underlined the role of key influencers involved in the stages before its drafting: Non-State Actors, mainly NGOs; others from the palliative care field and palliative care associations; individuals working in the area of human rights; Member States through their permanent missions in the WHO; and the WHO itself. This collaborative work was well described and centered on advocacy relating to deficient access to palliative care services and the promotion of a human rights focus linked to Universal Health Coverage.

In 2012, Panamá became a WHO Executive Board member for the first time and, with the support of Non-State Actors and other countries, led the proposal for a WHA Resolution on palliative care. As a result of the collaborative work done in advance by many of those involved and diplomatic actions carried out by Panamá, the adoption process itself was described by our interviewees as relatively straightforward and without major difficulties:

By the time the Resolution was presented in the Assembly, there were not many issues for people to reject, or to refuse, because a lot of groundwork had been done

### Processes of implementation

The “double responsibility” for the implementation of the Resolution (WHO Director-General and Member States) was clearly identified by the interviewees as important.

Post-Resolution actions undertaken by the WHO included the following: the appointment of a dedicated officer to focus on the implementation; the creation of advisory and technical cross-sectional working groups; and the development of strategies to collect data related to the provision of palliative care. The WHO role was identified as a key element and included promoting the idea of palliative care across disease and age groups, and also across departments and levels of the organization itself, plus generating tools for the implementation.

In the ‘Non-Communicable Diseases agenda’, there has been significantly more attention for palliative care. (…) WHO headquarters organized an extra half-day meeting that was dedicated to palliative care. (…) The Resolution has really helped mobilize more WHO resources for palliative care

The role of the Member States was described in the interviews, but with a remarkably skeptical perception about the (lack of) work done by most countries. Despite this, keeping palliative care on the various agendas of the WHO and Member States was identified as one of the key success factors achieved in the period following the adoption of the Resolution.

Funding and a lack of resources were seen as the most relevant hindrances, limiting the development of actions for the promotion of palliative care and the integration of services into national health systems. Cultural factors were also identified by those we interviewed as consequential for the implementation, hindering shared understanding and strategic clarity. The cultural factors cited included taboos and inhibitions relating to death and end of life, constraints on open communication when a person is dying, the desire to continue active treatment even when the direct benefits are limited, and highly varied public understandings about the goals and purposes of palliative care. Overall, the dearth of concrete proposals for implementation, the limited technical support offered in the early years, and the limited monitoring of strategies to assess its impact emerged as the major and more significant structural barriers to progressing the Resolution.

In contrast, the wide network of NGOs and palliative care associations working on advocacy at the national and international levels and providing technical support to governments and global agencies was identified as the main facilitator for the implementation. Our informants highlighted the cohesion of these stakeholders and the complementary work done by each of them in limiting the fragmentation of initiatives.

### Impact

Three years after the approval of the Resolution, most of the experts interviewed considered it too early to see clearly any resulting impact, especially given the lack of objective indicators for monitoring the implementation. However, this did not eclipse a perceived quickening in the movement to strengthen palliative care development around the world. The main identified impact of the Resolution was its use as an advocacy tool and its contribution to raising awareness about palliative care. This was seen to have legitimated efforts to include palliative care in public health and political strategies.

We've been able to use the Resolution as both an advocacy tool but also to be able to go to governments and say, look, you signed up to this at the WHA Assembly

Notwithstanding the progress on policy and advocacy for palliative care, some key experts pointed out the gaps between the development of relevant policies, the subsequent transfer to practice, the lack of progress in the delivery of education and in drug availability, and ultimately the final impact on people who could benefit.

There was agreement, however, that more impact had been seen in settings where palliative care is in an early stage of development, and that the influence of the WHO on implementation of the Resolution in low- and middle-income countries was a key example of progress.

## Discussion

Although we lack a set of indicators to assess the global impact of Resolution WHA67.19, we identified several efforts made to explore and to describe cognate actions carried out at different levels.

In 2015, the WHO *Non-Communicable Disease Country Capacity Survey* showed that a minority of countries include palliative care in their national policy for noncommunicable diseases, and reported that palliative care is least likely to have funding, compared with other noncommunicable disease services.^9^

In 2016, a civil society report pointed out that donor countries had not yet given adequate provision to facilitate the implementation process for the Resolution, although examples of progress on the integration of palliative care into the health policy were cited (India, Colombia, Romania); progress in palliative care education was illustrated (Jordan, Panama, Morocco); and improvements in drug availability (Mexico) and in rolling out palliative care services (Ethiopia) were presented.^10^

Also in 2016, the WHO Secretariat reported a range of actions by Member States, the WHO headquarters, and regional and country office focused on access to oral morphine and essential medicines; the integration of palliative care into global disease control and health system plans; and information about guidance, tools, and training programs on palliative care.^11^ The same year, the WHO published a practical manual on how to plan and implement palliative care services integrated into existing health care services.^12^

In 2017, an edited collection *Building Integrated Palliative Care Programs and Services* was published by a group of activists aiming to assist governments and providers to build and strengthen palliative care provision.^13^

However, a global survey of palliative care in 2017 concluded that a mere 14% of the world population has access to the highest levels of palliative care provision, while 53% live in countries where palliative care delivery is localized to a significant degree and lacks sufficient integration with the wider health and social care system to achieve high coverage.^1^

Since the approval of the Resolution, palliative care has been integrated in subsequent WHO or WHA resolutions,^14–16^ but these are nonbinding in character, making for difficulties in global implementation and in the assessment of impact on national policies.

## Conclusions

Key experts evaluate the short-term effects of WHA67.19 as positive for its policy implications and the use that palliative care champions are making of it. If it is not possible to identify a tangible transfer to people who need palliative care, the follow-up to the Resolution is expected to achieve improvements in palliative care development around the world, a process in which the role of Non-State Actors, the WHO, and Members States working together will be crucial.

## Appendix

**Appendix Table A1.** People Interviewed^*^

Name	Surname	Organization
Liz	Gwyther	Worldwide Hospice Palliative Care Alliance (WHPCA)
Cynthia	Goh	Asia Pacific Hospice Palliative Care Network (APHPCN)
Philip	Larkin	European Association for Palliative Care (EAPC)
Liliana	De Lima	International Association for Hospice & Palliative Care (IAHPC)
Sharon	Baxter	Canadian Hospice Palliative Care Association (CHPCA)
Stephen	Connor	Worldwide Hospice Palliative Care Alliance (WHPCA)
Marie-Charlotte	Bouësseau	World Health Organization (WHO)
Julia	Downing	International Children's Palliative Care Network (ICPCN)
Emmanuel	Luyirika	African Palliative Care Association (APCA)
Eric L.	Krakauer	Harvard Medical School
		World Health Organization (WHO)
Diederik	Lohman	Human Rights Watch
Katherine P	Pettus	International Association for Hospice & Palliative Care (IAHPC)
Mary V	Callaway	International Association for Hospice & Palliative Care (IAHPC)
Kathleen	Foley	Neurologist in the Pain and Palliative Care Service at Memorial Sloan-Kettering Cancer Center (MSKCC)
M R	Rajagopal	Pallium India
Gaspar	Da Costa	Health Ministry of Panamá
Patricia	Bonilla	Asociación Latinoamericana de Cuidados Paliativos (ALCP)
Silvana	Luciani	Pan American Health Organization (PAHO)
James	Cleary	Pain and Policy Studies Group
Jorge	Corrales	Counselor to the Permanent Mission to the United Nations and other International Organizations in Geneva

^*^ Speaking in their own name, not on behalf of their organizations.
